# Acupuncture for lumbar disc herniation: Protocol for a systematic review and meta-analysis

**DOI:** 10.1097/MD.0000000000019117

**Published:** 2020-02-28

**Authors:** Sheng Yuan, Chuyu Huang, Yuanyue Xu, Dong Chen, Lei Chen

**Affiliations:** a The First Affiliated Hospital of Jinan University; b Second Clinical College of Medicine; c Acupuncture and Rehabilitation Clinical Medical College, Guangzhou University of Chinese Medicine; d Traditional Chinese Medicine Hospital of Guangdong Province, Guangzhou, China.

**Keywords:** lumbar disc herniation, acupuncture, systematic review, meta-analysis, protocol

## Abstract

**Background:**

As development of society and change of modern life style, the prevalence of lumbar disc herniation (LDH) has been increasing. Being a major cause of low back pain, sciatica and radicular leg pain, LDH imposes a heavy burden on both individual and society. Because of high surgically intervene rate, non-invasive (non-surgical) treatments are recommended for most cases. Acupuncture has the advantages of low risk, good effect and low cost which has been proven that could alleviate pain while physical therapy plays a major role in the treatment of LDH in the vast majority of countries. The aim of this systematic review is to evaluate the effectiveness and safety of acupuncture for LDH.

**Methods:**

RCTs on ACU treating LDH will be searched from the following databases: PubMed, Web of science, EmBase, Cochrane Library, China National Knowledge Infrastructure, Wanfang data, from their inception to May 2020. The primary outcomes are verbal rating scale and functional disability. Two reviewers will independently exclude substandard articles and extract eligible data. The risk of bias will be assessed using the Cochrane Handbook 5.1.0 for Systematic Reviews of Interventions. Egger test will be used to assess the reporting bias. Heterogeneity will be evaluated by the I^2^ statistic and Q test. We will conduct the meta-analysis using Stata V12.0 to evaluate the effectiveness of ACU for LDH. In case of high heterogeneity, sensitivity analysis of different items and subgroup analysis will be performed. The Grading of Recommendations Assessment, Development, and Evaluation System will be used to assess the quality of evidence.

**Results:**

The results of this review will be submitted to a journal for publication.

**Conclusion:**

This proposed systematic review will evaluate the effectiveness and safety of acupuncture for LDH.

**Registration:**

PROSPERO **(**registration number CRD42019148272).

## 1 Introduction

Lumbar disc herniation (LDH), also called herniated lumbar disc, whose definition is localized displacement of disc material beyond the normal margins of the intervertebral disc space resulting in pain, weakness, or numbness in a myotomal or dermatomal distribution,^[1]^ which is a major cause of sciatica, low back pain and radicular leg pain.^[2]^ The cardinal symptoms of LDH include low back pain radiating to posterior aspect of thigh and leg, numbness and paraesthesia in respective dermatome and weakness, depressed reflexes in corresponding myotome.^[3]^ A variety of studies showed that the most common location of LDH was at L4-L5 (40%), followed by L5-S1 (36.8%), whereas L2-L3, and L1-L2 accounted for (13.2%) each.^[4]^ Prevalence of Recurrent LDH is reported to be 5% to 18%.^[5]^ A sizable percentage of clinically relevant herniation attack between the ages of 30 years and 50 years, but can also occur in adolescent and older people.^[6]^ Studies showed that 24%-27% of the asymptomatic have LDH.^[7,8]^ As development of society and change of modern life style, the incidence of LDH has become higher and higher. Although the exact etiology is not fully known, factors like mechanical loading, occupation, autoimmune, genetic have been related to increase the risk of LDH.^[9]^ Middle age working male are more prone to develop LDH because of long distance driving, labour, carpentry, barber and office working.^[9]^ LDH not only reduces quality of life, but also incurs considerable medical expenses. It has been shown that LDH imposes a heavy economic burden on both individual and society.^[10]^

The treatments of LDH could be roughly divided into 3 parts, non-invasive treatments, minimally invasive procedures and surgery.^[2]^ Study has reported a surgically intervene rate of 24% and 8% of patients who underwent surgery had complications among which nearly 50% of the complications being serious.^[11]^ It has been proven that current surgical techniques are less invasive compared to the past, while significant problems still exist in terms of effectiveness, safety, and cost.^[11]^ Experts suggest non-invasive (non-surgical) treatments to be a first-line choice for most cases.^[6]^ Pharmacological therapy (non-steroidal anti-inflammatory drugs) is one of the most used non-invasive treatments,^[1]^ while it also increases the risk of complications of cardiovascular disease, even with acute usage.^[12]^

**Figure 1:**
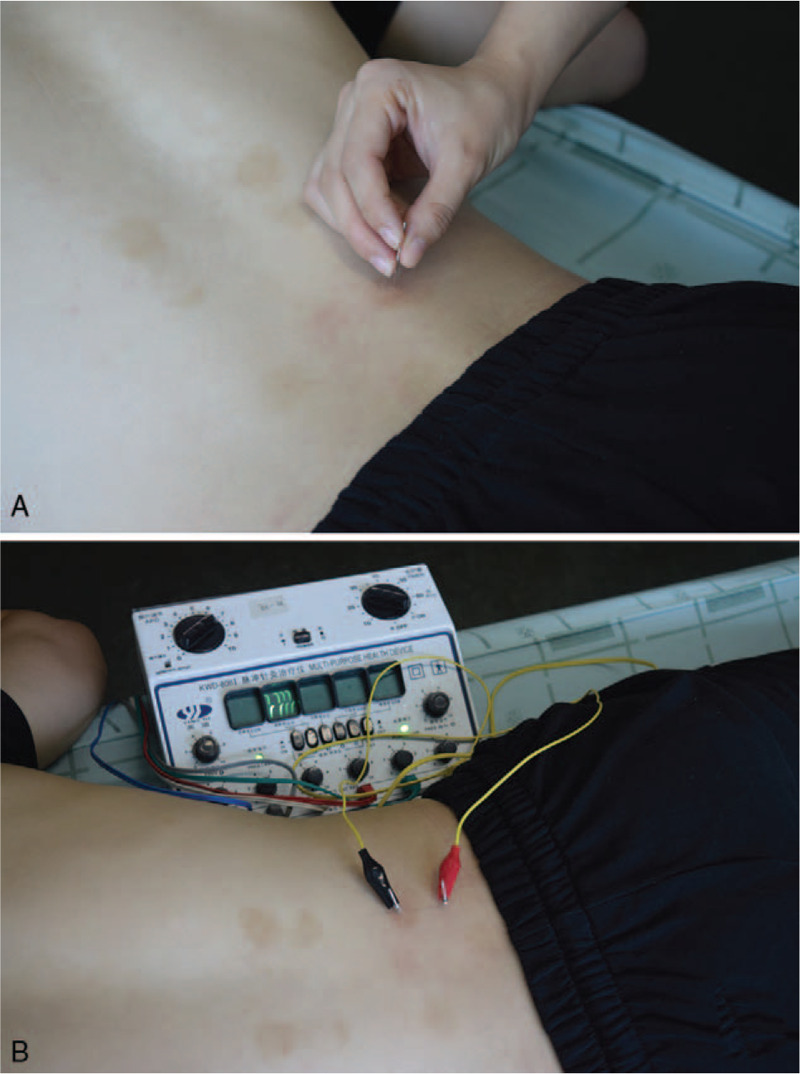
Manual ACU and electroacupuncture.

Acupuncture (ACU), a therapy with long history in China, has been reported to have significant effects on reducing pain and improving quality of life among back-pain sufferers.^[13]^ ACU analgesia improved the noxious descending inhibitory controls and pain gate mechanism and, therefore, helped the patients’ pain levels.^[13]^ Consequently, the number of patients with LDH has increased^[14]^ because the change in life-style happened over the past few decades and there is no update in previous systematic reviews on this topic, so a new systematic review of the literature is needed. Most of the available systematic reviews and meta-analyses compare the effectiveness of surgery with conservative treatments for LDH. As mentioned above, ACU is an advisable treatment for LDH, we will compare ACU with other treatments in adult patients with symptomatic LDH. This systematic review and meta-analysis will evaluate the effectiveness of ACU for LDH, including manual ACU and electroacupuncture (Fig. 1). We deeply believe that a well-conducted systematic review and meta-analysis is important to benefit the widespread of ACU and to better inform clinicians, therapists and patients about the effectiveness of ACU.

## 2 Methods

### 2.1 Study registration

The agreement is based on the Preferred Reporting Project (PRISMA-P) statement for system evaluation and meta-analysis. This study has been registered on PROSPERO (www.crd.york.ac.uk/ PROSPERO) and the registration number is CRD42019148272.

### 2.2 Inclusion criteria for study selection

#### 2.2.1 Type of study

We will include randomized controlled trials (RCTs) had been published of LDH treated with ACU. Only articles in English or Chinese will be selected whereas animal studies will be excluded.

#### 2.2.2 Type of participants

LDH adult subjects (≥18 years) without gender restriction confirmed by magnetic resonance imaging (MRI) or CT will be included in our studies. Patients may be accompanied by symptoms like sciatica or low back pain. Studies that include patients with specific or systematic diseases (infection, tumor, osteoporosis, fracture, structural deformity, ankylosing spondylities, radicular syndrome or cauda equina syndrome, etc)^[15]^ or pregnancy will be excluded.

#### 2.2.3 Type of interventions

The experimental group should be applied individual treatment with ACU (including manual ACU and electroacupuncture) or together with pharmacotherapies. Studies which combine ACU treatments with pharmacotherapy are required to use the same pharmacotherapy in both the experimental and the control groups. There will be no limit on the duration or frequency of the treatments. The control group will include non-ACU treatments (including sham ACU, pharmacotherapies, surgery, acetylcholinesterase inhibitors, among others).

#### 2.2.4 Type of outcome measures

The primary outcomes of interest will be verbal rating scale (VRS) and functional disability. The secondary outcomes of interest will include McGill pain questionnaire, quality of life, muscle tension, muscle strength and recurrence rate.

### 2.3 Electronics searches

#### 2.3.1 Search strategy

We will search the following electronic databases: PubMed, Web of science, EMBase, Cochrane Library, China National Knowledge Infrastructure (CNKI), Wanfang data, Chinese Biomedical Literature database (CBM) and VIP. We anticipate to search the databases from their inception to May 2020. The strategy will be created according to the Cochrane handbook guidelines. Searching terms will be used as MeSH terms and free-text. The specific search strategy for PubMed is shown in Table 1.

**Table 1:**
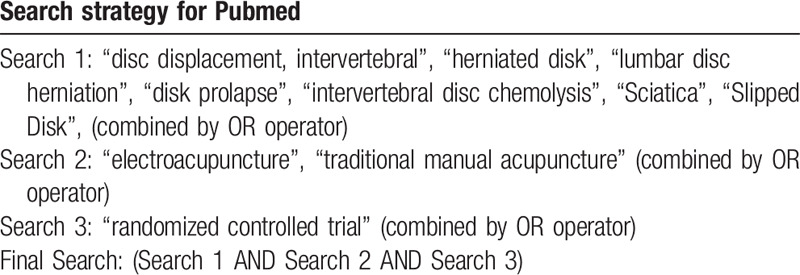
Electronic search procedure.

### 2.4 Statistical analysis

#### 2.4.1 Identification of studies

**Figure 2:**
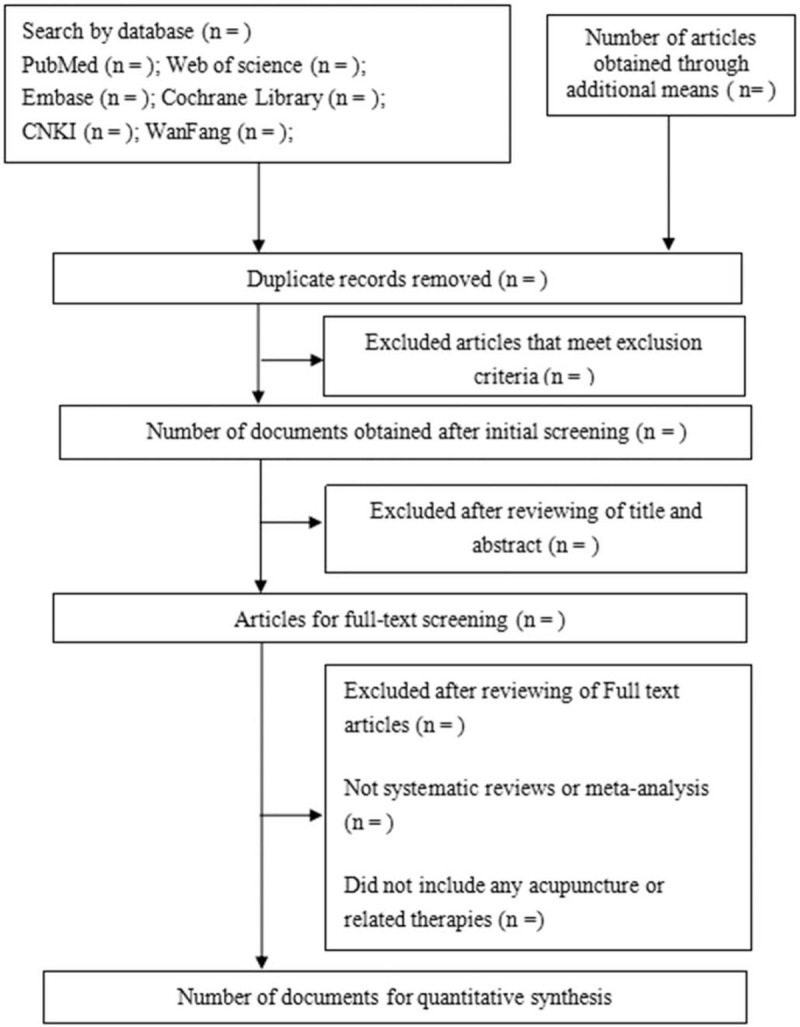
Preferred Reporting Items for Systematic Reviews and Meta-Analyses (PRISMA) flow chart.

The retrieved literature will be imported into an EndNote library to count and eliminate the duplicate. Two reviewers (Y-YX, C-YH) will independently scan the title and abstract to exclude substandard articles whereas the rest go on to be read in full text. The extracted information is as follows: author, year of publication, country, study design, sample size, participants, ACU intervention, control intervention, outcomes, and adverse events. A third reviewer (SY) will participate in the extraction and discussion if the first two have controversial information. The primary selection process is shown in a Preferred Reporting Items for Systematic Reviews and Meta-Analyses (PRISMA) flow chart (Fig. 2).

#### 2.4.2 Dealing with missing data

In case of the situation that the article data is missing, we will try to contact corresponding authors by email if necessary. If we fail to obtain valid data, we may assess the potential impact in the article by the existing data. The study will be excluded if that cannot be analyzed.

#### 2.4.3 Risks of bias

Two reviewers will access the risk of bias employing the Cochrane Handbook 5.1.0 for Systematic Reviews of Interventions, which comprises 7 items: random sequence generation, allocation concealment, blinding of participants and personnel, blinding of outcome assessment, incomplete outcome data, selective reporting, and other sources of bias.^[16]^ The assessment of risks of bias will be classified into three levels: low risk of bias, high risk of bias and unclear risk of bias.^[17]^

#### 2.4.4 Assessment of reporting bias

If a sufficient number of studies are included in, Egger test will be used to assess the reporting bias. The statistical significance exists when *P* < .01 and demonstrates the analysis in the Discussion part.

#### 2.4.5 Assessment of heterogeneity

Heterogeneity can be evaluated by the I^2^ statistic and Q test.^[18]^ We will use Stata V.12.0 software to evaluate the heterogeneity of the included studies by the test statistics. We will indicate continuous results with the effect estimation, which is mean difference with 95% CIs. I^2^ < 25% indicates no significant heterogeneity, I^2^ = 25% to 50% is considered as moderate heterogeneity, and heterogeneity will be considered substantive if I^2^ > 50%, as *P* < .05 will be considered as indicating significant differences.^[19]^ If there exists heterogeneity, data will be analyzed with the random effects models, otherwise, the fixed effects models will be adopted.

#### 2.4.6 Sensitivity analysis

In case of high heterogeneity, we will conduct a sensitivity analysis of different items as following: research types, age differences, gender differences, quality of research, treatment duration, treatment frequency and heterogeneity qualities.

#### 2.4.7 Subgroup analysis

We will conduct a subgroup analysis among gender, age, racial, duration and frequency of treatment and ACU stimulus gradient. Quantitative analysis or qualitative analysis is decided to take or not according to the quantity of extracted information. If the extracted data is insufficient for quantitative analysis, qualitative analysis will be carried out to assess the robust of our data.

#### 2.4.8 Grading the quality of evidence

We will use the Grading of Recommendations Assessment, Development, and Evaluation (GRADE) method for evaluation.^[19]^ The quality of evidence for RCTs will be graded as 4 levels as follow: very low, low, moderate, or high.

### 2.5 Patient and public involvement

This research won’t involve any patient or public.

### 2.6 Ethics and dissemination

Our expected goal is to publish the result of this study in a peer-reviewed journal.

## 3 Discussion

Due to the herniation of the nucleus pulposus, LDH is characterized by the corresponding segmental nerve symptoms such as lumbar pain and sciatica.^[20]^ ACU stimulates blood circulation at corresponding acupoints thereby relieving pain, which is also the basis for ACU to treat pain caused by LDH.^[21]^ From the current point of view, there are no sufficient evidence for ACU therapy of LDH as the relevant comprehensive research is still blank. The study aims to explore the potential relationship of treatment and safety between the effectiveness of ACU and LDH by conducting a systematic review and meta-analysis. We hope the results of this study will provide guidance for physician's decision-making and the development and update of guidelines.

## Author contributions

**Data curation:** Lei Chen.

**Formal analysis:** Sheng Yuan.

**Funding acquisition and validation:** Dong Chen.

**Methodology:** Sheng Yuan, Yuanyue Xu.

**Writing – original draft:** Sheng Yuan, Yuanyue Xu.

**Writing – review and editing:** Sheng Yuan, Chuyu Huang.
